# Supplementary material for: A universal surface functionalization technique to chemically enhance live microbial cells
Source: Mol Syst Biol. 2026 Mar 16;22(6):962–78. doi: 10.1038/s44320-026-00202-z (PMC13230988; doi:10.1038/s44320-026-00202-z)
Supplement: Supplementary file 2 — Appendix [file 44320_2026_202_MOESM2_ESM.pdf]

Appendix for “A universal surface functionalization technique to chemically enhance live microbial cells“

Table of contents

- Appendix Figure S1: Quantification of single-cell fluorescence in fluorophore-functionalized cells.
- Appendix Figure S2: Quantification of surface functionalization efficiency and cell viability.
- Appendix Figure S3: Estimation of growth rates for cultures treated with increasing crosslinker concentrations.
- Appendix Figure S4: Flow cytometer fluorescence quantification for fluorophore-functionalized strains.
- Appendix Figure S5: Regrowth dynamics for crosslinker treated strains.
- Appendix Figure S6: Treatment of E. coli cells with sodium periodate and aniline.
- Appendix Figure S7: Benchmarking of biotin-sulfo-NHS-croslinker.

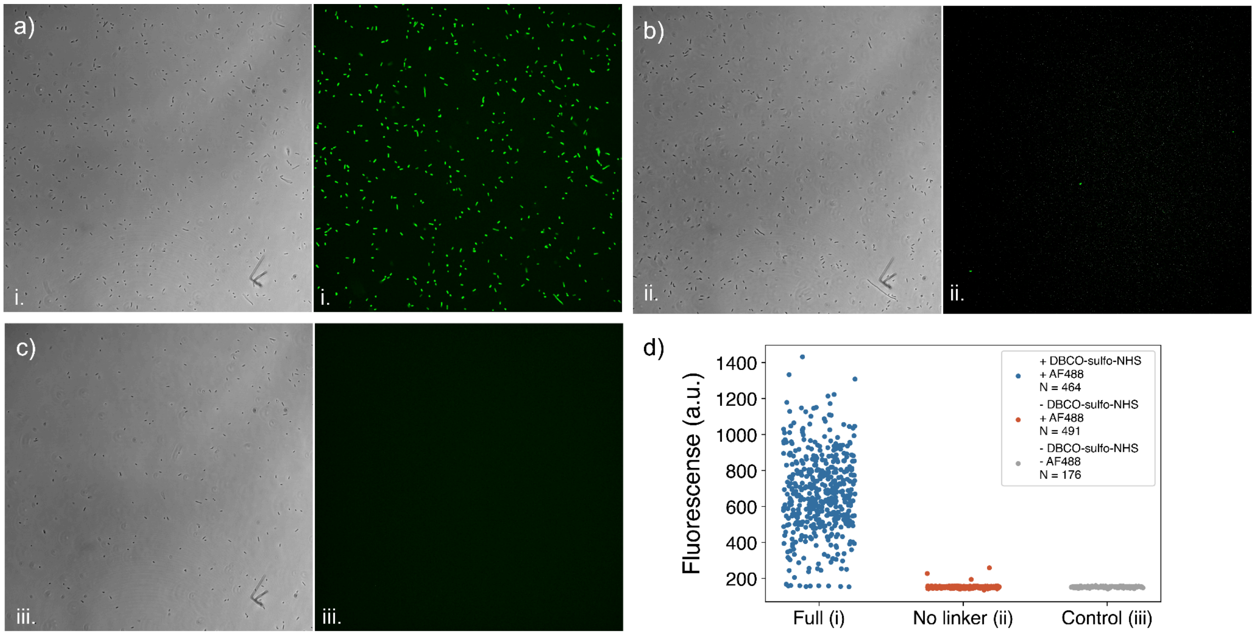

Appendix Figure S1: Quantification of single-cell fluorescence in fluorophore-functionalized cells. (a-c) Illustrative transmitted light and green channel images for treatments with (i) DBCO-sulfo-NHS and Alexa Fluor 488, (ii) Only Alexa Fluor 488, and (iii) No treatment. (d) Quantification of fluorescence intensity from raw pixel values. Cells were segmented in the image using Ilastik-1.4.0-OSX and the mean pixel value for each cell was plotted. The number of cells analyzed in each condition is shown as N.

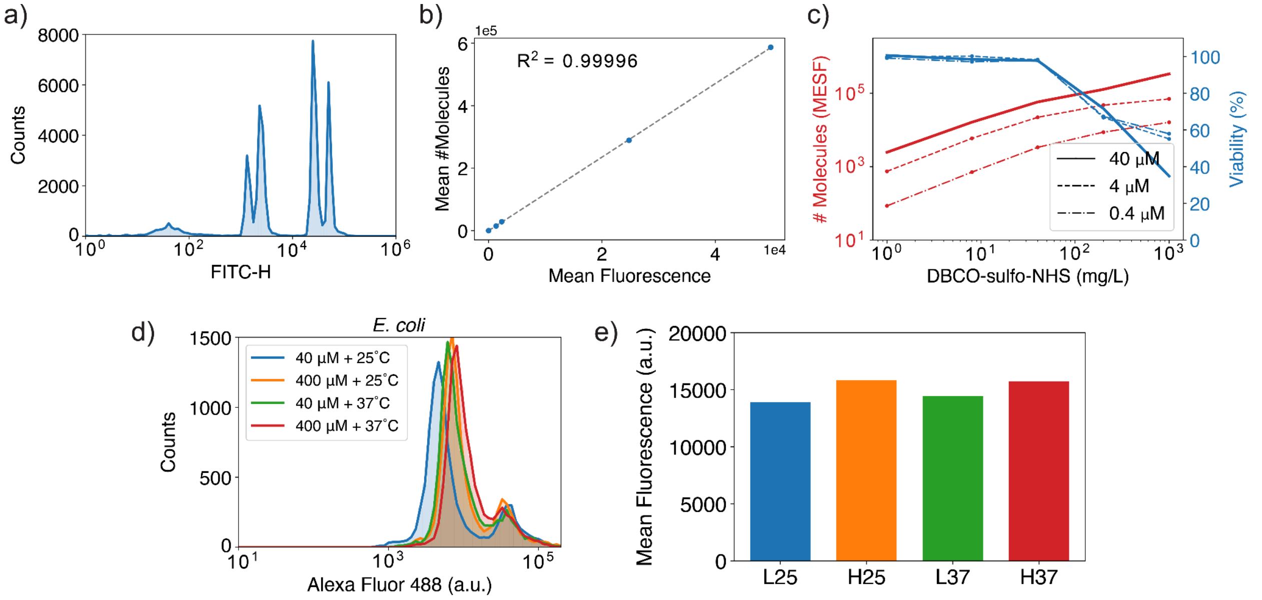

Appendix Figure S2: Quantification of surface functionalization efficiency and cell viability. (a) Fluorescence quantification of flow cytometry calibration beads from Quantum™ Alexa Fluor® 488 MESF kits. The analyzed sample was a mixture of 5 solutions of beads containing different amounts of fluorophores. (b) Calibration curve based on measured fluorescence for each of the 5 solutions of calibration beads and their reported mean number of fluorophores. (c) Influence of DBCO-sulfo-NHS (x-axis) and Alexa Fluor 488 azide (legend) concentration on the number of molecules loaded onto E. coli cell surfaces and cell viability. While changes in the crosslinker and fluorophore concentration change the number of molecules on the cell surface, only changes in the crosslinker concentration affect cell viability. (d) Effect of incubation temperatures (25 ºC or 37 ºC) and Alexa Fluor 488 azide concentration (40 or 400 μM) on fluorescence acquired by cells. Increasing neither temperature nor azide molecule concentration led to significantly higher cell fluorescence. (e) Bar plot showing mean fluorescence of histograms in (d) for conditions L25 (40 μM + 25 ºC), H25 (400 μM + 25 ºC), L37 (40 μM + 27 ºC) and H37 (400 μM + 37 ºC). E. coli cells in (d) and (e) were treated with 40 mg/L of DBCO-sulfo-NHS. Each point represents a single biological sample and mean fluorescence intensity values were averaged across 10000 flow cytometry events per sample.

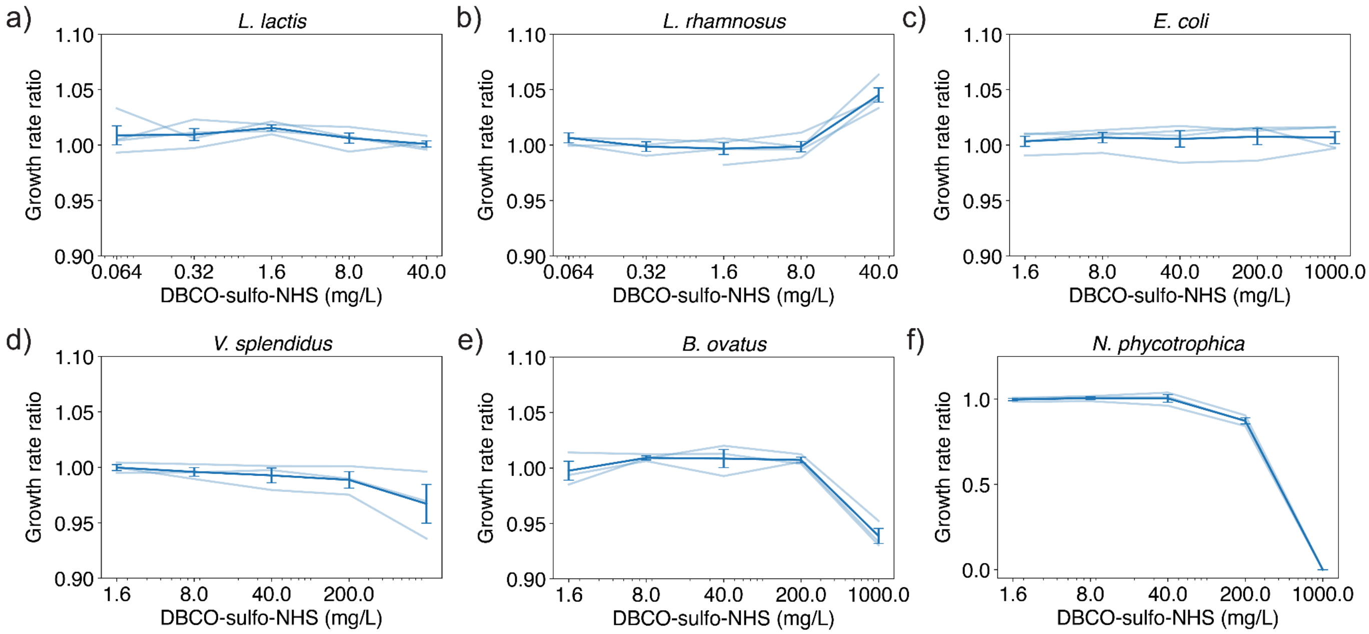

Appendix Figure S3: Estimation of growth rates for cultures treated with increasing crosslinker concentrations. Data was recorded using the regrowth experiment methodology. Growth rate ratio was calculated as the ratio between the growth rate for a specific condition and the growth rate of its paired untreated control. Error bars represent SEM across 4 biological replicates for L. lactis, L. rhamnosus, and E. coli, and 3 biological replicates for all other strains. Individual biological replicates are shown as semi-transparent curves.

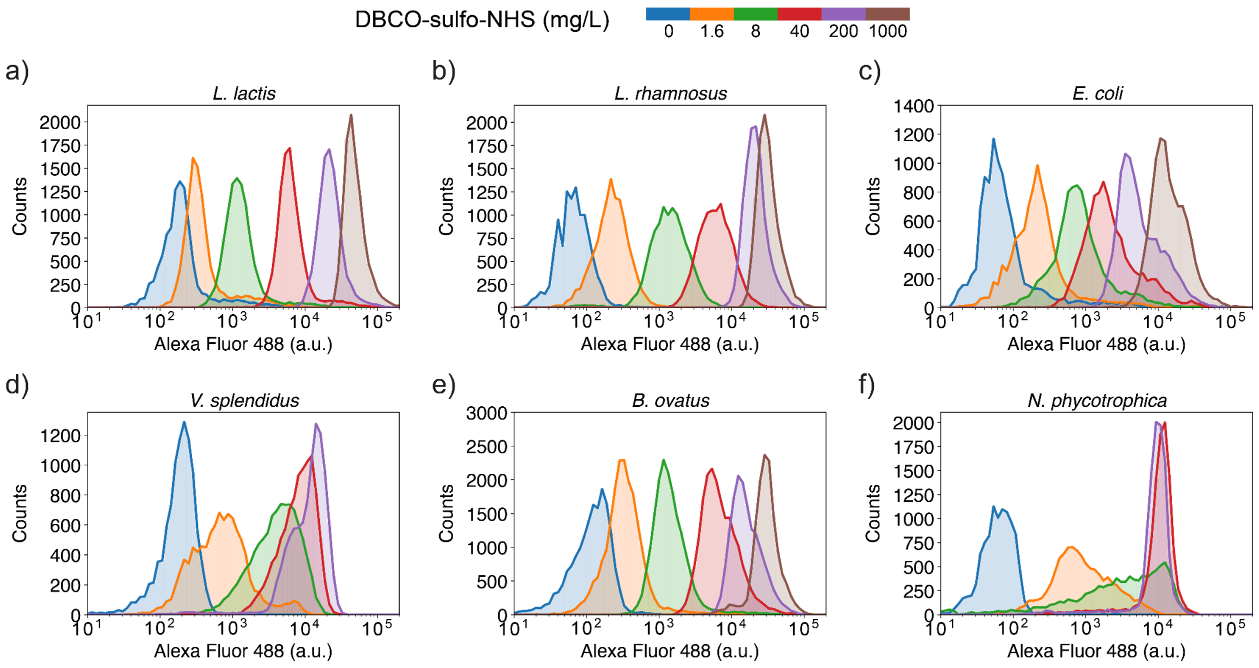

Appendix Figure S4: Flow cytometer fluorescence quantification for fluorophore-functionalized strains. The data displayed is from a single biological replicate for illustration purposes.

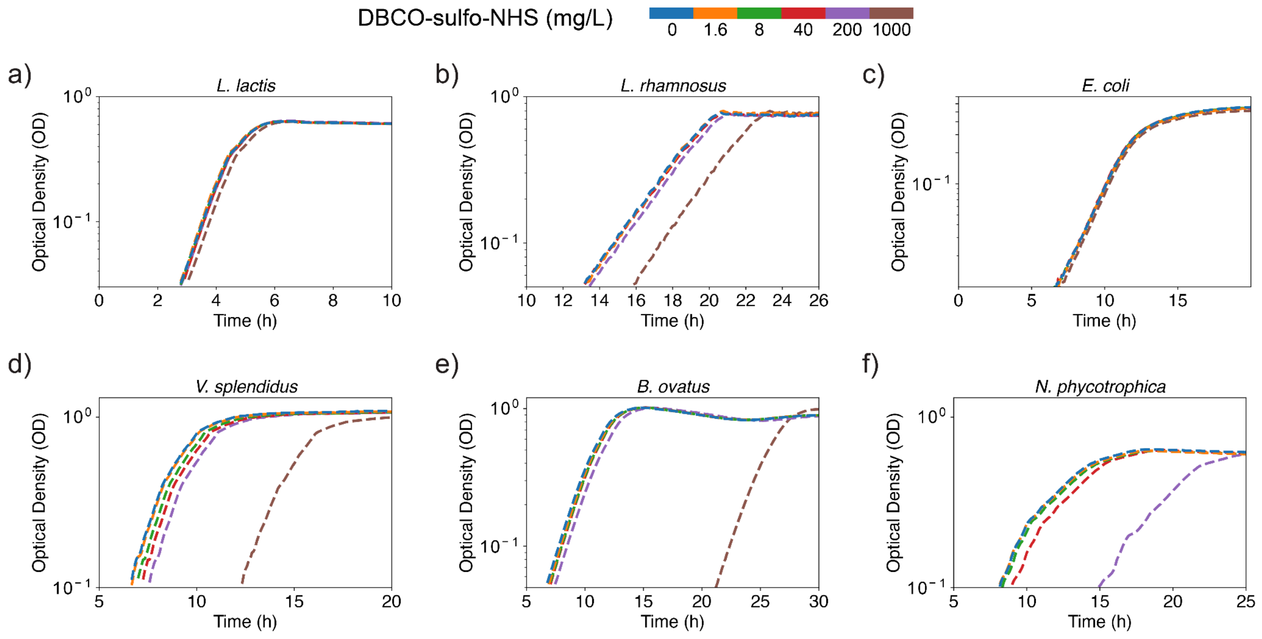

Appendix Figure S5: Regrowth dynamics for crosslinker treated strains. The data displayed is from a single biological replicate for illustration purposes. Growth delays were used to calculate cell viability shown in the main text.

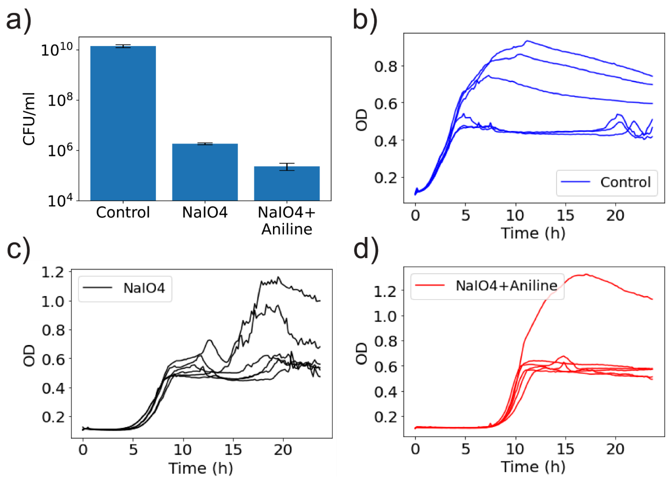

Appendix Figure S6: Treatment of E. coli cells with sodium periodate and aniline. (a) CFU counts per ml for E. coli cells treated with 0.5 mM of sodium periodate for 20 minutes followed by incubation with 25mM Aniline for 4h (NaIO4 + Aniline) or not (NaIO4). Control condition was not exposed to any chemicals (Control). CFU counts for Control, NaIO4 and NaIO4 + Aniline conditions were 57, 135, and 9, respectively, across 6 biological replicates. Error bars represent standard error of counts assuming a Poisson distribution. (b-d) Regrowth dynamics of cells in the same treatment conditions as (a) showing significant delays in growth. Each line represents an independent biological replicate.

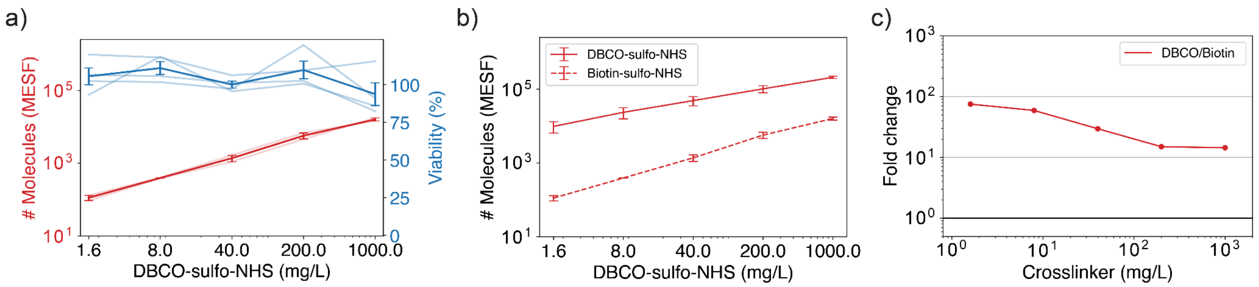

Appendix Figure S7: Benchmarking of biotin-sulfo-NHS-croslinker. (a) Quantification of efficiency and viability trade-off across different crosslinker concentrations for the treatment of E. coli cells. Viability was quantified by the re-growth method (4 biological replicates) and efficiency was estimated with flow cytometry and converted to MESF units using calibration beads (2 biological replicates, 10000 events per sample). (b) Comparison between the biotin and DBCO crosslinkers' efficiency curves. (c) Fold change in the number of molecules achieved with functionalization using the DBCO crosslinker versus the biotin crosslinker (DBCO/biotin). The black horizontal line represents a fold change of 1 and thus equal efficiency. Error bars represent standard error of the mean in all panels.
