# Supplementary material for: A universal surface functionalization technique to chemically enhance live microbial cells
Source: Mol Syst Biol. 2026 Mar 16;22(6):962–78. doi: 10.1038/s44320-026-00202-z (PMC13230988; doi:10.1038/s44320-026-00202-z)
Supplement: Supplementary file 3 — Table EV1 [file 44320_2026_202_MOESM3_ESM.docx]

Table EV1: Comparison of different surface functionalization methods.

| **Functionalization method** | | **Strains evaluated** | **Commercial availability of reagents** | **Molecules coupled** | **Regrowth viability** | **Efficiency (MFI fold change)** | **Efficiency (MESF)** | **Protocol duration (h)** | **Reference(s)** |
| --- | --- | --- | --- | --- | --- | --- | --- | --- | --- |
| Chemical | DBCO-sulfo-NHS | Escherichia coli, Lactobacillus lactis, Lactococcus rhamnosus, Bacteroides ovatus, Vibrio Splendidus, Neptunomonas phycotrophica | All reagents available | Fluorophore, DNA, protein | 88 ± 6% | 110x | 210k | 2 – 3 | This work |
| Chemical | Biotin-sulfo-NHS | Escherichia coli | All reagents available | Fluorophore | 93 ± 7% | 40x | 15k | 2 – 3 | This work |
| Chemical | Biotin-sulfo-NHS | Lactobacillus casei, Escherichia coli, and Bacillus coagulans | All reagents available | Fluorophore, Antibody | ~50% (estimated) | 4x (estimated) | 4k (estimated) | 1 – 2 | 5 |
| Chemical | Hydrazine-aldehyde coupling | Escherichia coli, Bacillus subtilis, Saccharomyces cerevisiae, Shewanella oneidenses, Azotobacter vinelandii, Rhodospirillum rubrum, Synechocystis sp. PCC6803, Chlamydomonas reinhardtii | All reagents available | DNA | < 0.01% | 13x (*) | 26k (*) | 12 – 16 | 25 |
| Biological | Metabolic labeling (azide-functionalized amino acid) | Escherichia coli | All reagents available | Biotin | No data | 10x | No data | 16 | 41 |
| Biological | Metabolic labeling (azide-functionalized sugar) | Escherichia coli, Staphylococcus aureus | All reagents available | DNA | No data | 6x | No data | 10 – 12 | 42 |
