# Supplementary figures and images for: A universal surface functionalization technique to chemically enhance live microbial cells
Source: Mol Syst Biol. 2026 Mar 16;22(6):962–78. doi: 10.1038/s44320-026-00202-z (PMC13230988; doi:10.1038/s44320-026-00202-z)

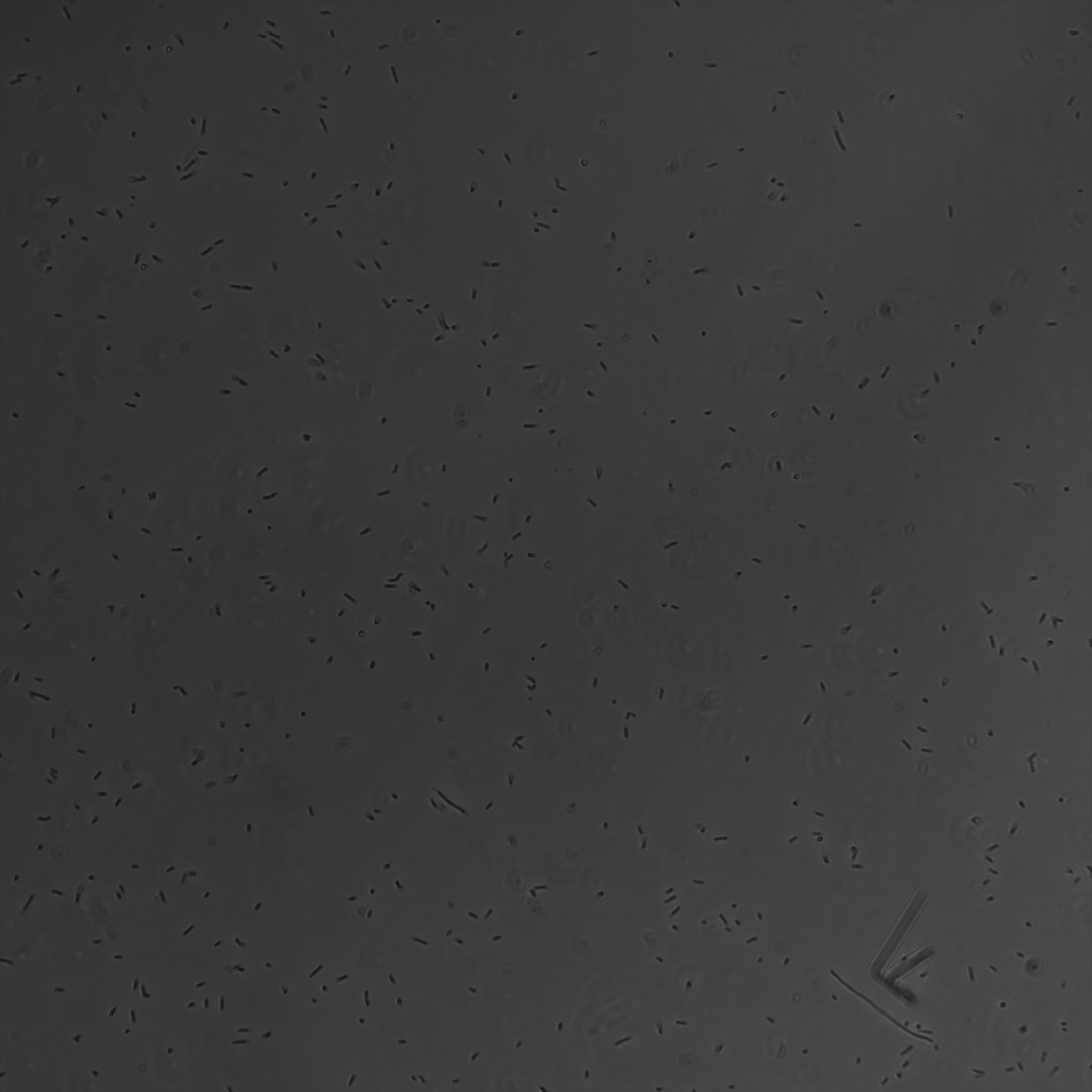

Supplement: Supplementary file 5 — Source data Fig. 2 [file 44320_2026_202_MOESM5_ESM.zip › SD figure 2/2A/brightfield_subpanel-ii.TIF]

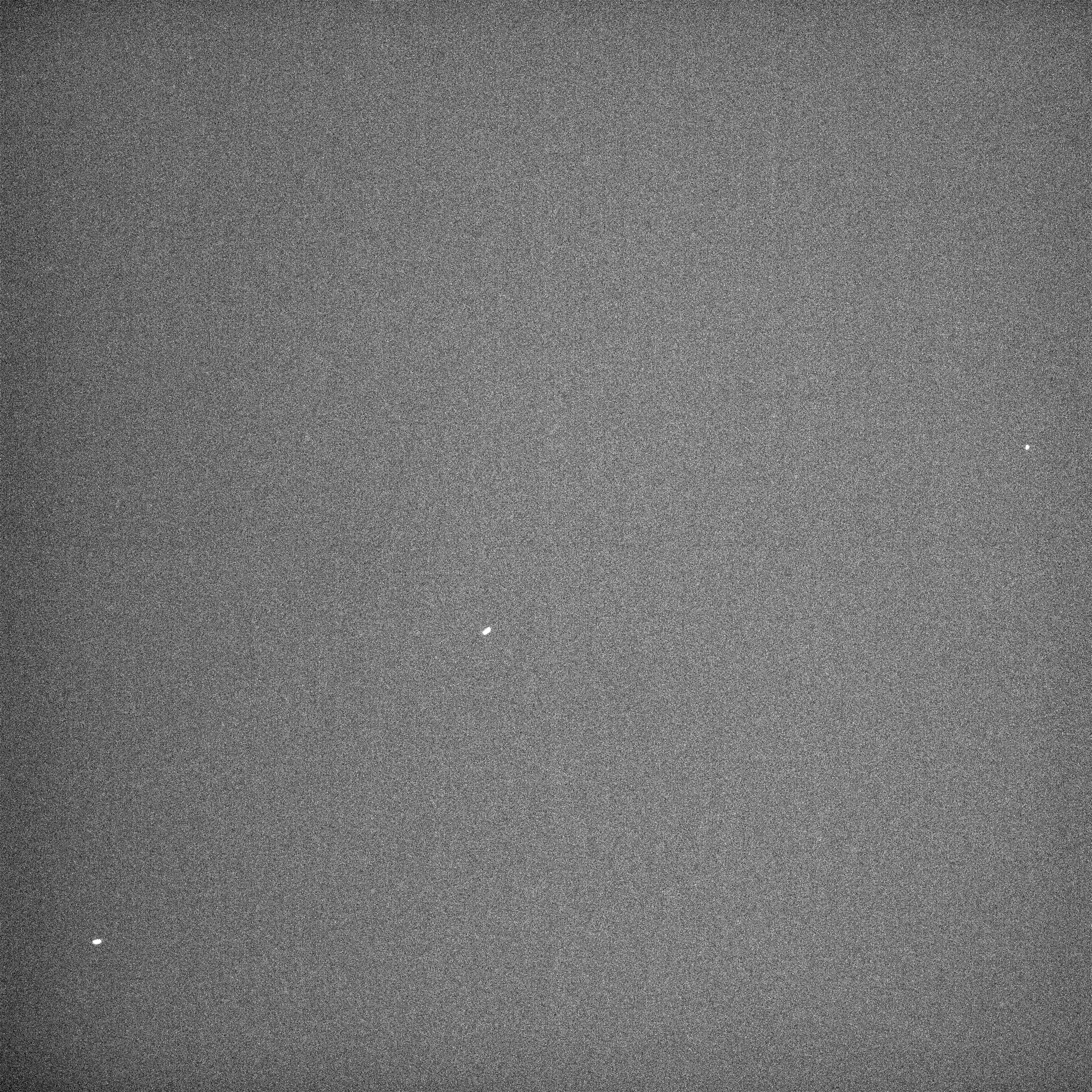

Supplement: Supplementary file 5 — Source data Fig. 2 [file 44320_2026_202_MOESM5_ESM.zip › SD figure 2/2A/fluorescence-subpanel-ii.TIF]

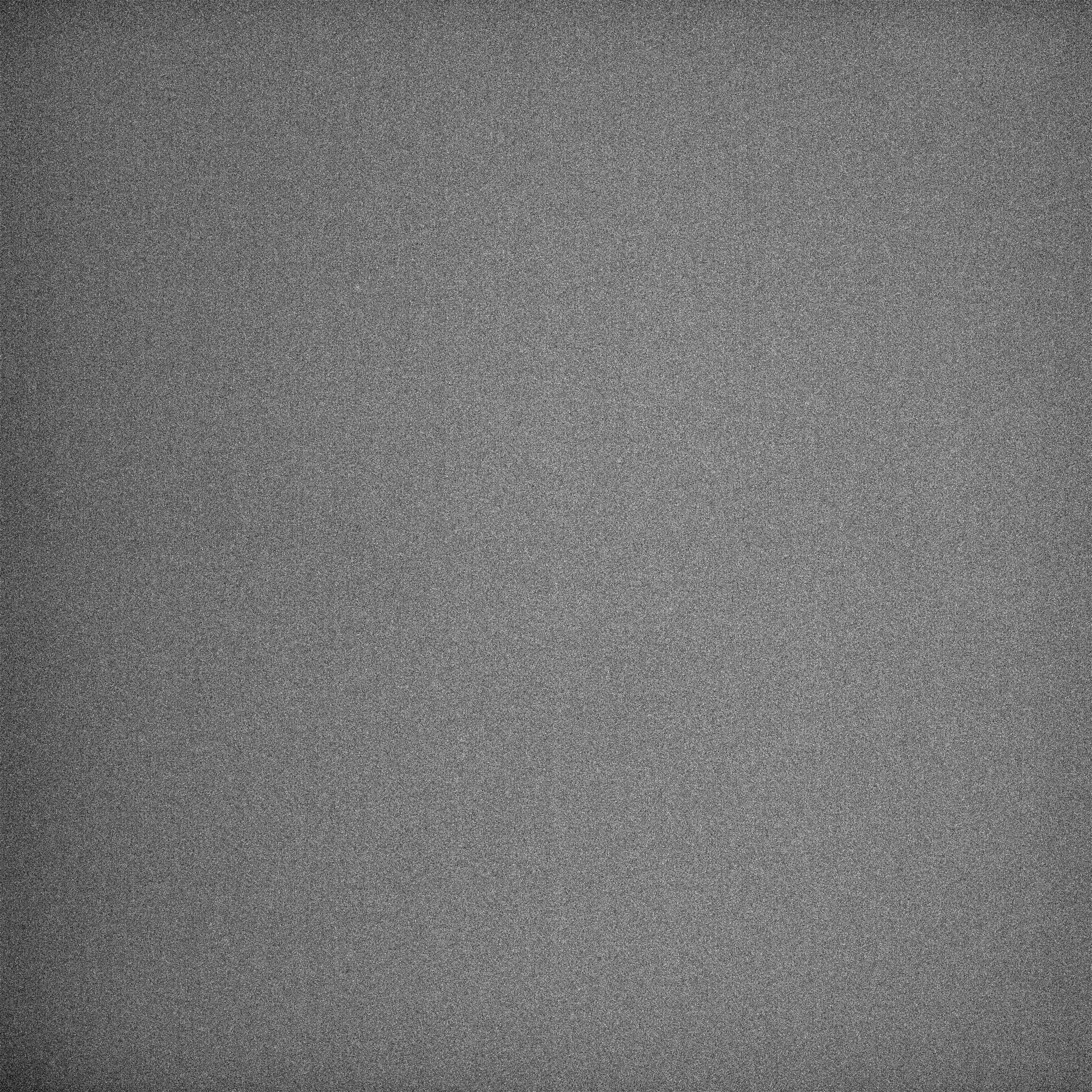

Supplement: Supplementary file 5 — Source data Fig. 2 [file 44320_2026_202_MOESM5_ESM.zip › SD figure 2/2A/fluorescence-subpanel-iii.TIF]

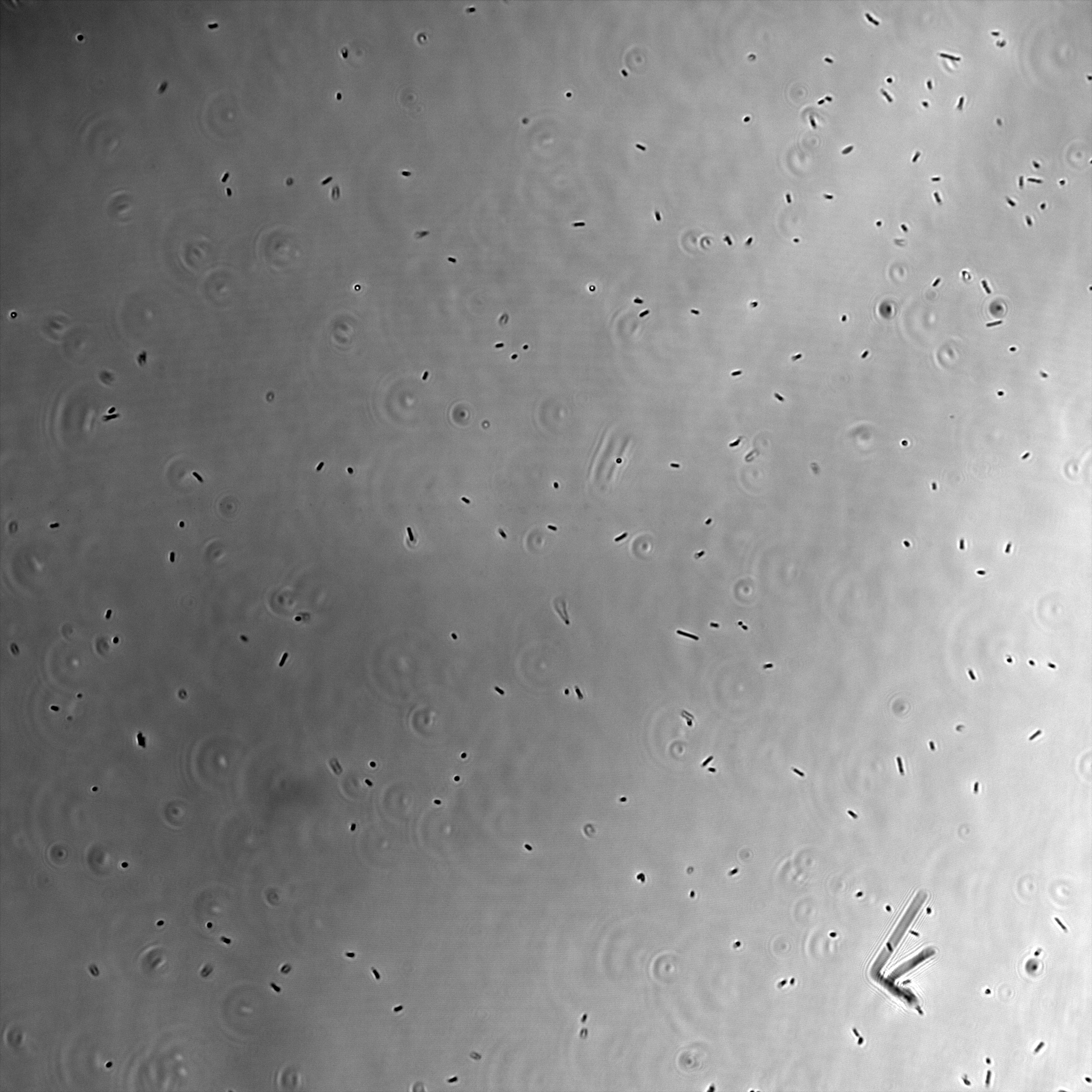

Supplement: Supplementary file 5 — Source data Fig. 2 [file 44320_2026_202_MOESM5_ESM.zip › SD figure 2/2A/brightfield_subpanel-iii.TIF]

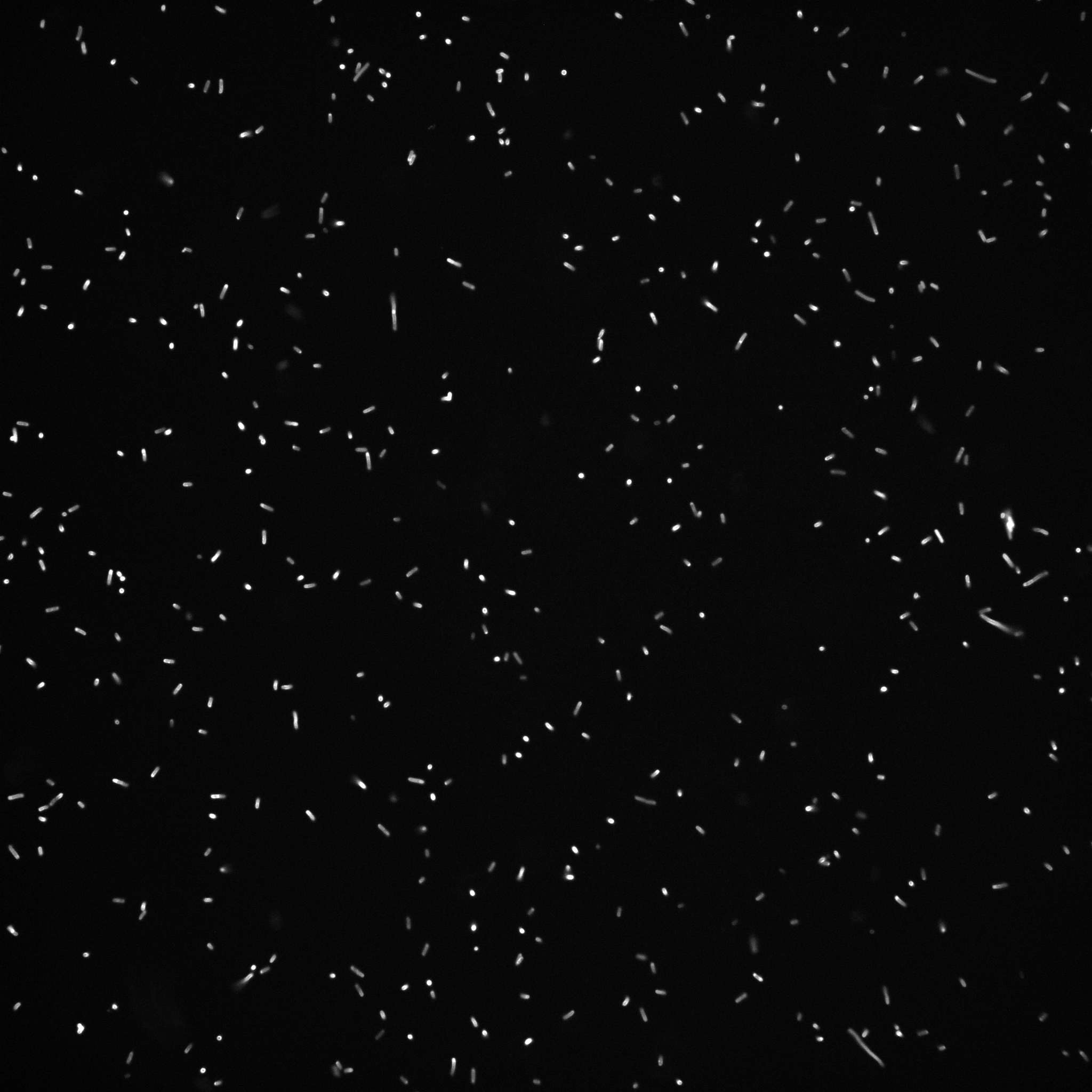

Supplement: Supplementary file 5 — Source data Fig. 2 [file 44320_2026_202_MOESM5_ESM.zip › SD figure 2/2A/fluorescence-subpanel-i.TIF]

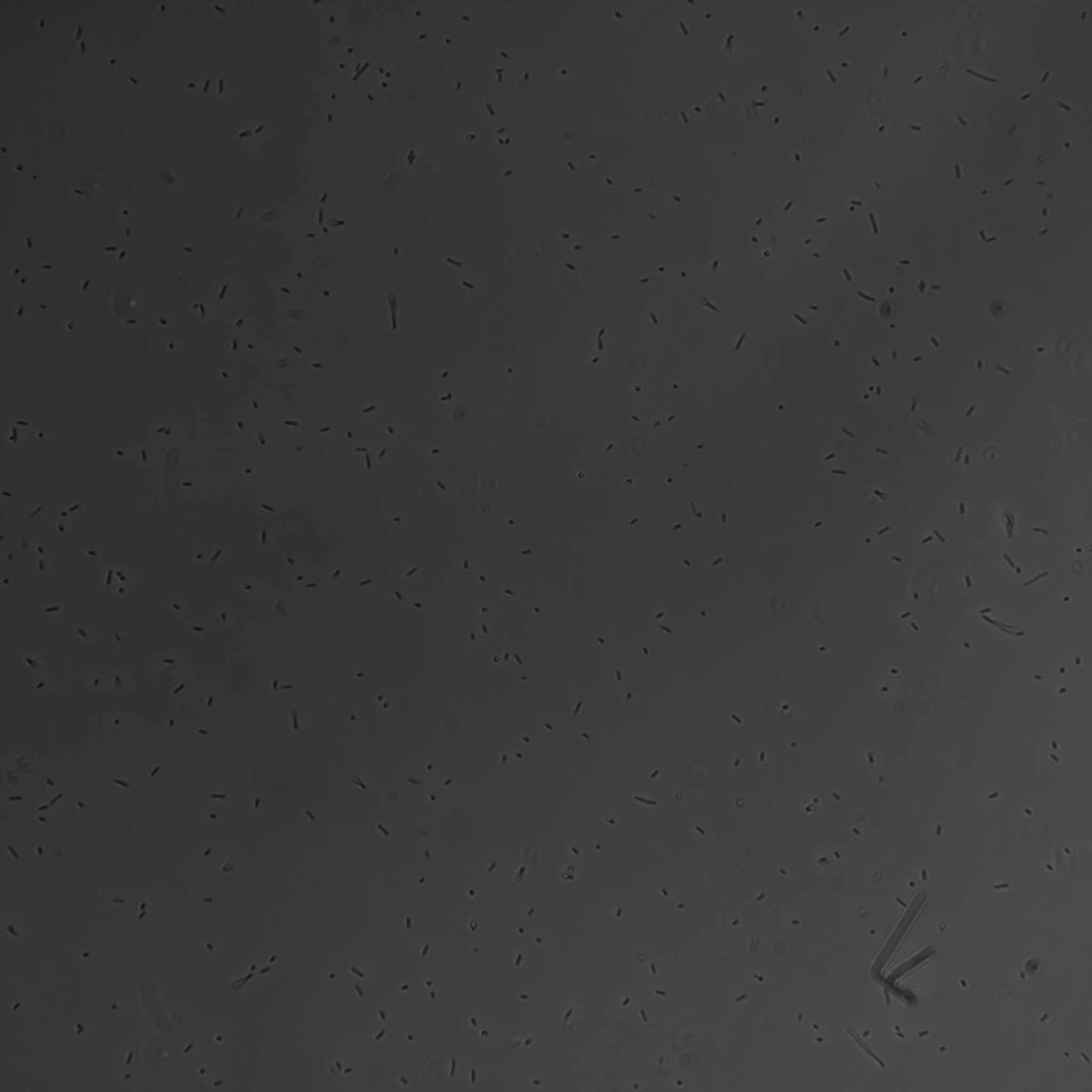

Supplement: Supplementary file 5 — Source data Fig. 2 [file 44320_2026_202_MOESM5_ESM.zip › SD figure 2/2A/brightfield_subpanel-i.TIF]

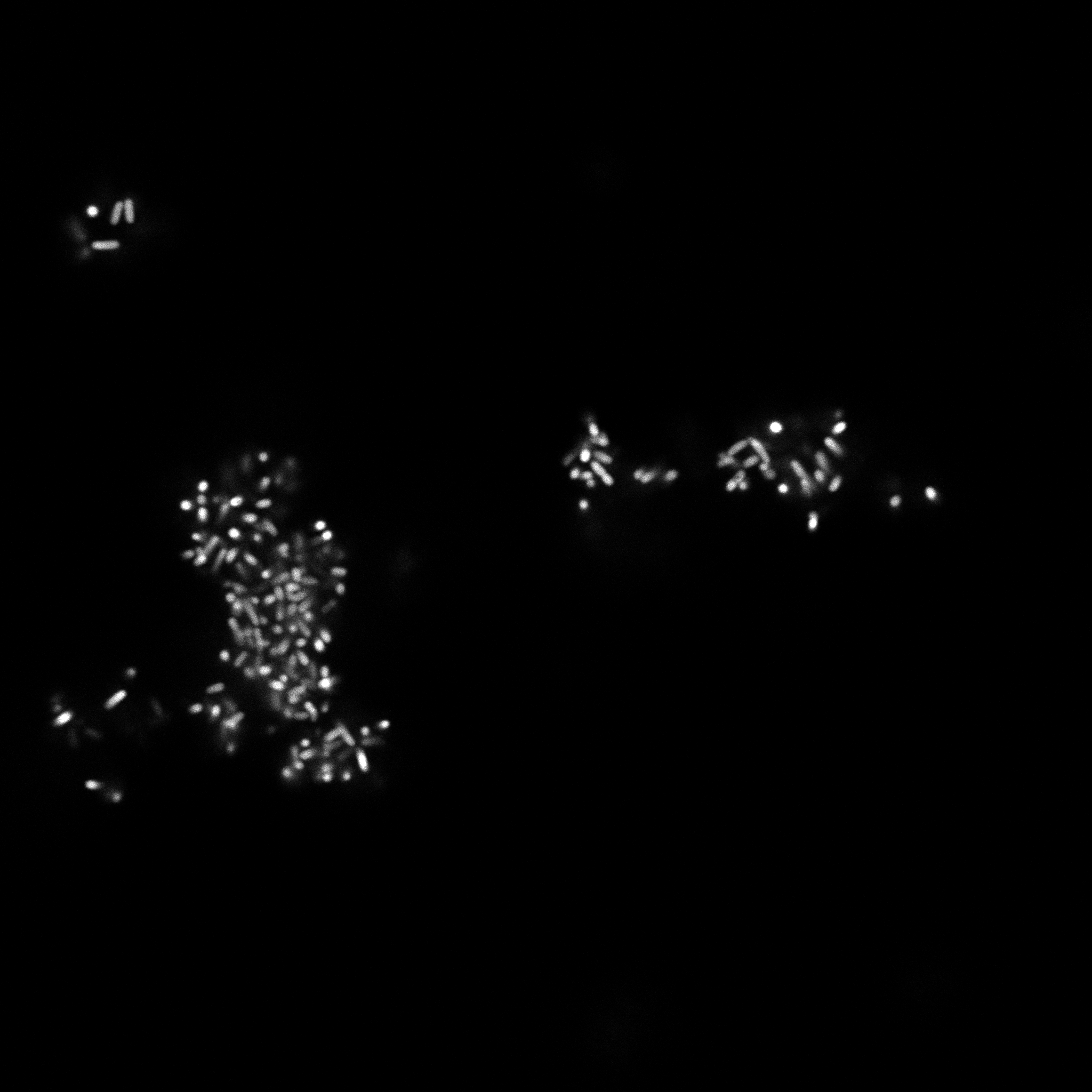

Supplement: Supplementary file 6 — Source data Fig. 5 [file 44320_2026_202_MOESM6_ESM.zip › SD figure 5/5G/checkerboard_2.tif]

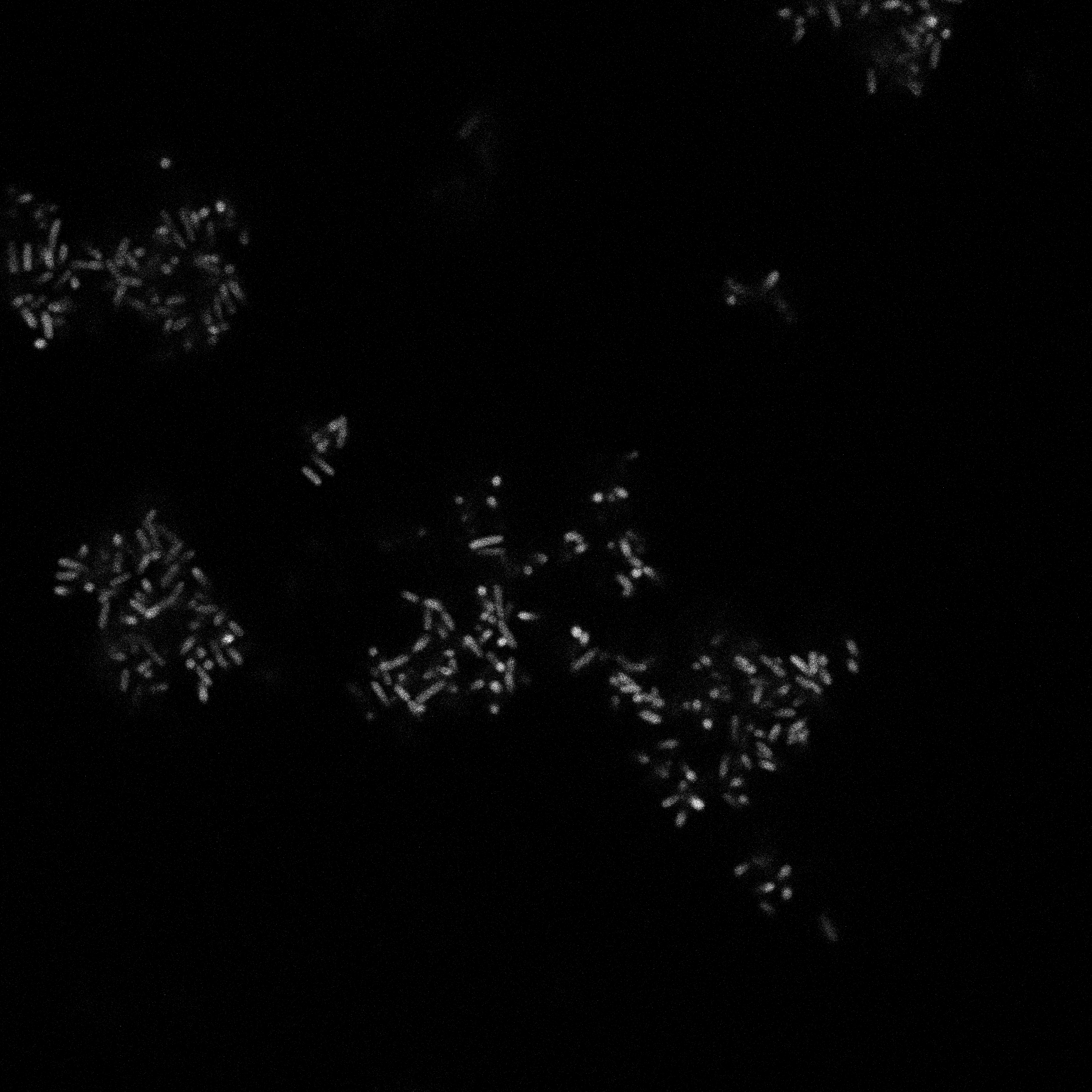

Supplement: Supplementary file 6 — Source data Fig. 5 [file 44320_2026_202_MOESM6_ESM.zip › SD figure 5/5G/checkerboard_3.tif]

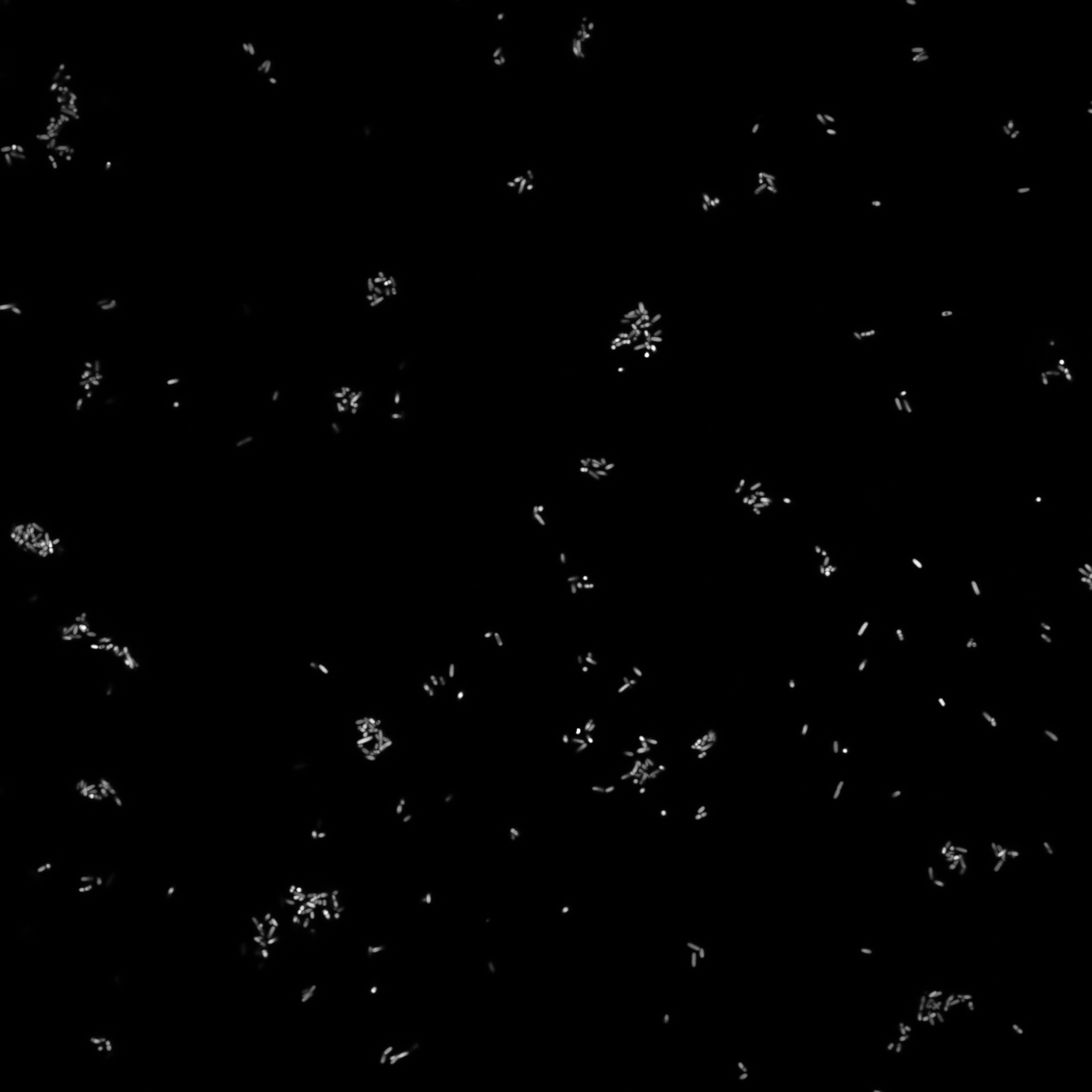

Supplement: Supplementary file 6 — Source data Fig. 5 [file 44320_2026_202_MOESM6_ESM.zip › SD figure 5/5G/checkerboard_1.tif]

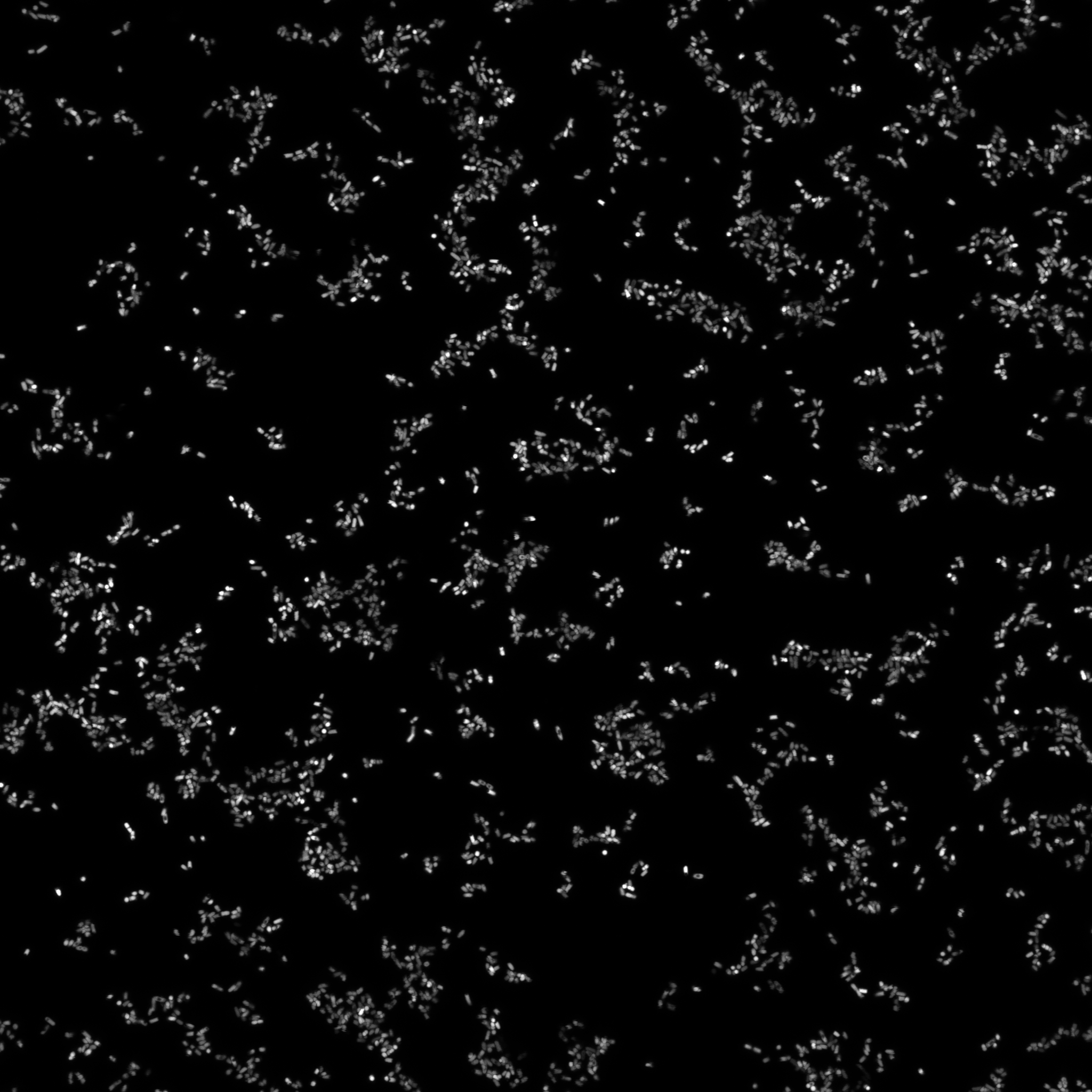

Supplement: Supplementary file 6 — Source data Fig. 5 [file 44320_2026_202_MOESM6_ESM.zip › SD figure 5/5G/random_3.tif]

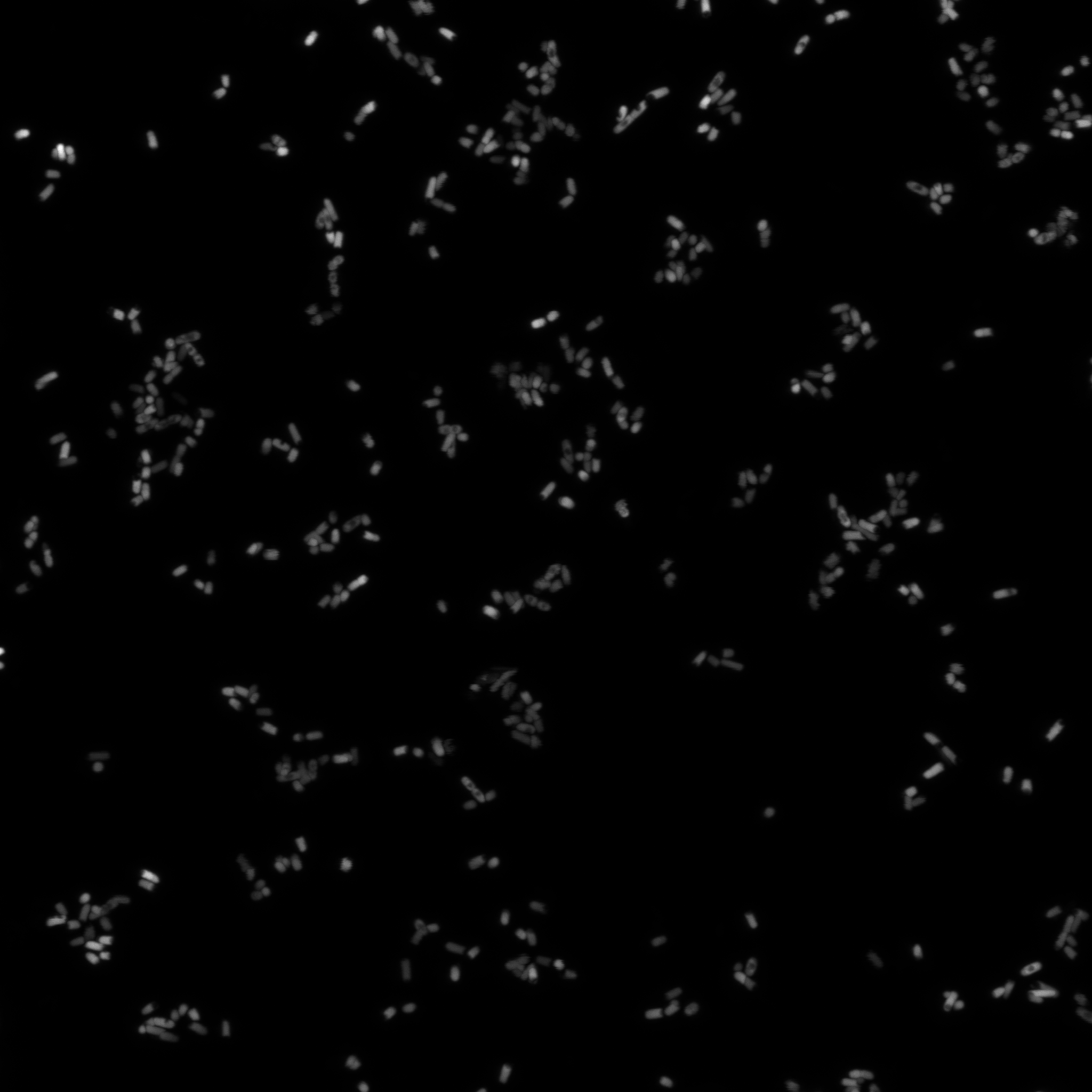

Supplement: Supplementary file 6 — Source data Fig. 5 [file 44320_2026_202_MOESM6_ESM.zip › SD figure 5/5G/random_2.tif]

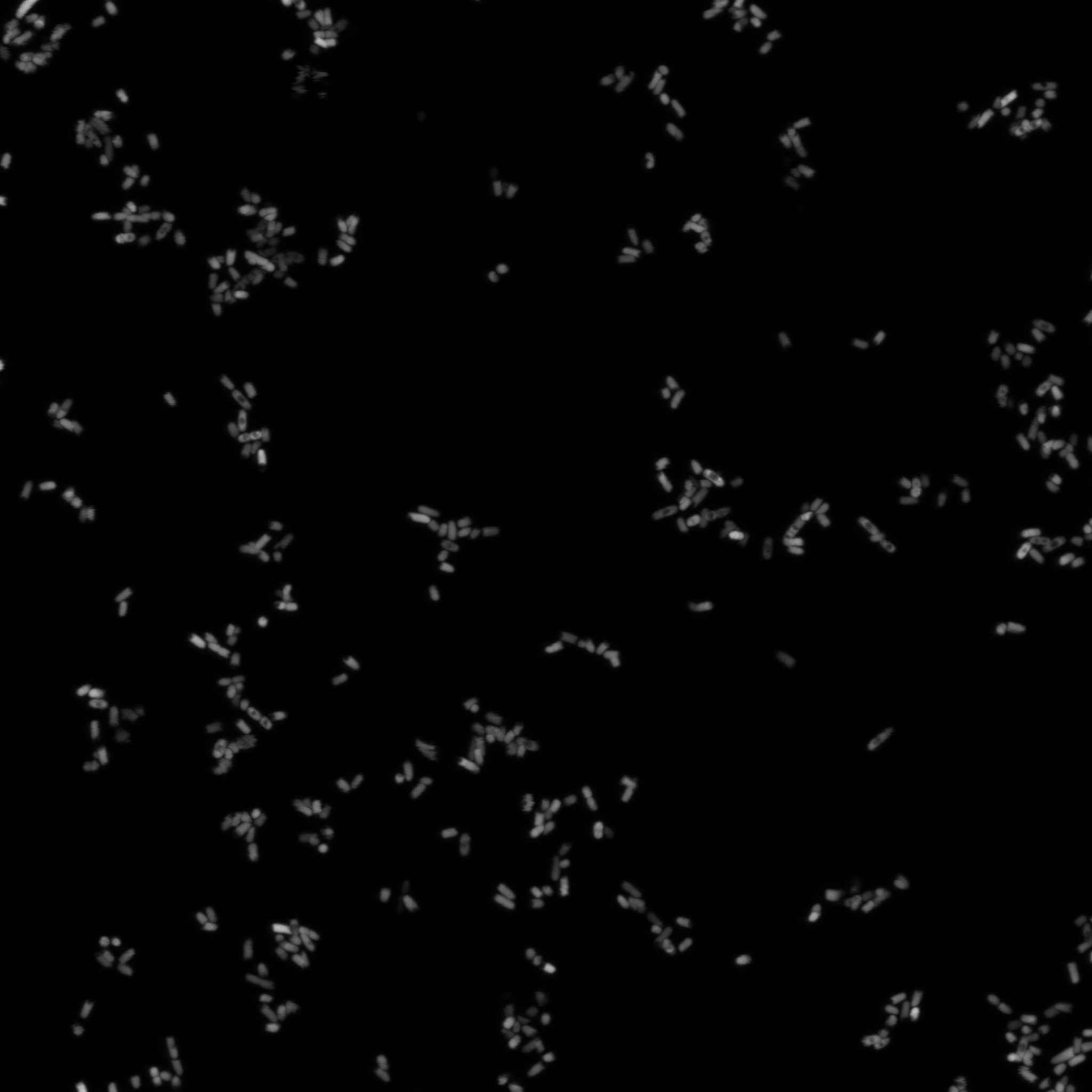

Supplement: Supplementary file 6 — Source data Fig. 5 [file 44320_2026_202_MOESM6_ESM.zip › SD figure 5/5G/random_1.tif]

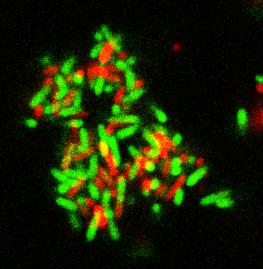

Supplement: Supplementary file 6 — Source data Fig. 5 [file 44320_2026_202_MOESM6_ESM.zip › SD figure 5/5F/checkerboard_example.tif]

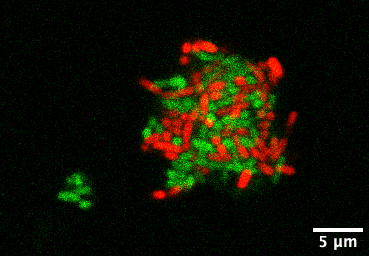

Supplement: Supplementary file 6 — Source data Fig. 5 [file 44320_2026_202_MOESM6_ESM.zip › SD figure 5/5H/timelapse-3.png]

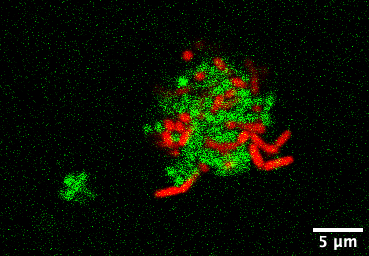

Supplement: Supplementary file 6 — Source data Fig. 5 [file 44320_2026_202_MOESM6_ESM.zip › SD figure 5/5H/timelapse-2.png]

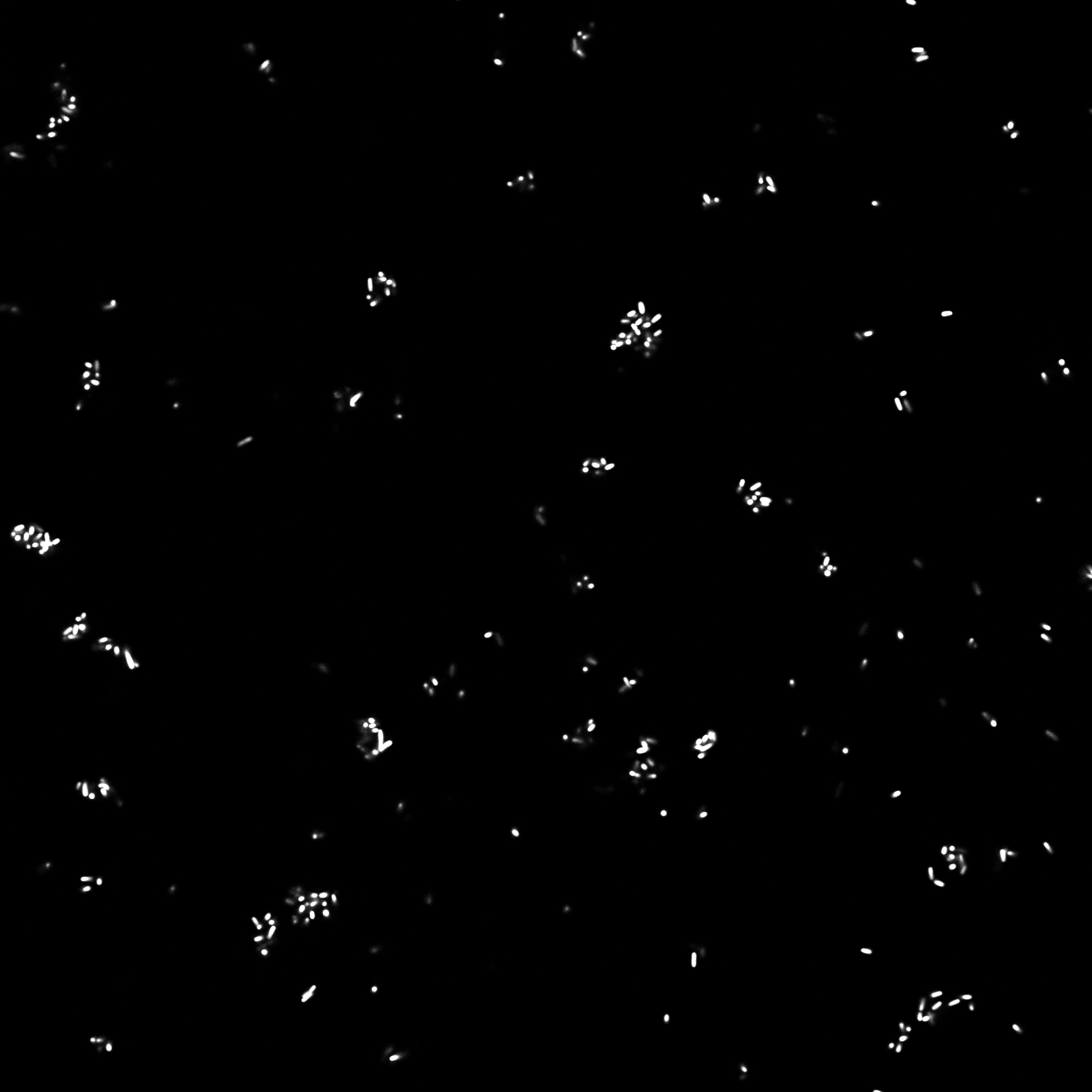

Supplement: Supplementary file 6 — Source data Fig. 5 [file 44320_2026_202_MOESM6_ESM.zip › SD figure 5/5H/timelapse.tif]

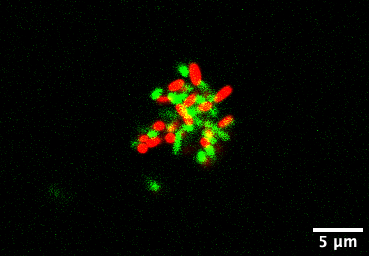

Supplement: Supplementary file 6 — Source data Fig. 5 [file 44320_2026_202_MOESM6_ESM.zip › SD figure 5/5H/timelapse-1.png]

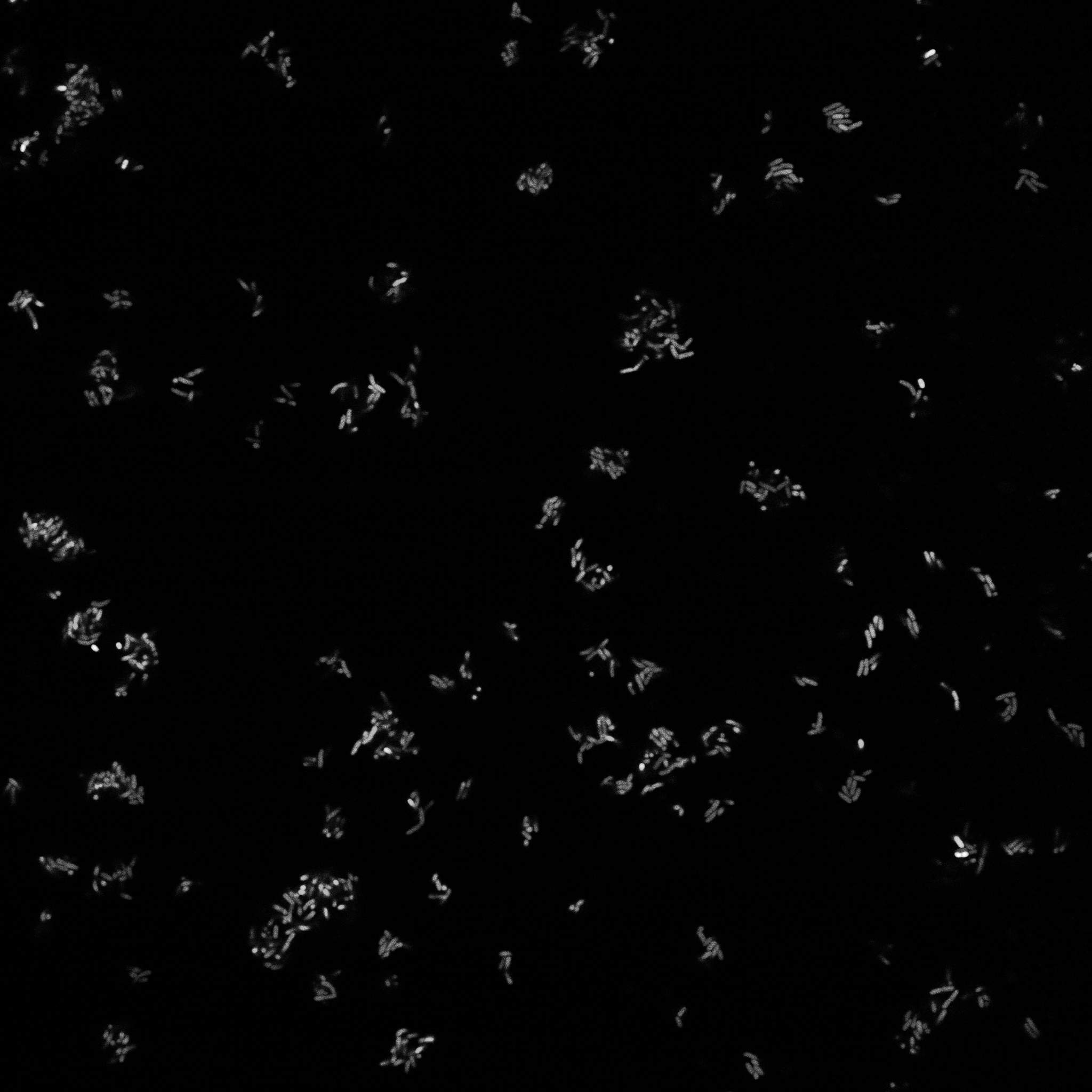

Supplement: Supplementary file 6 — Source data Fig. 5 [file 44320_2026_202_MOESM6_ESM.zip › SD figure 5/5H/t6.tif]

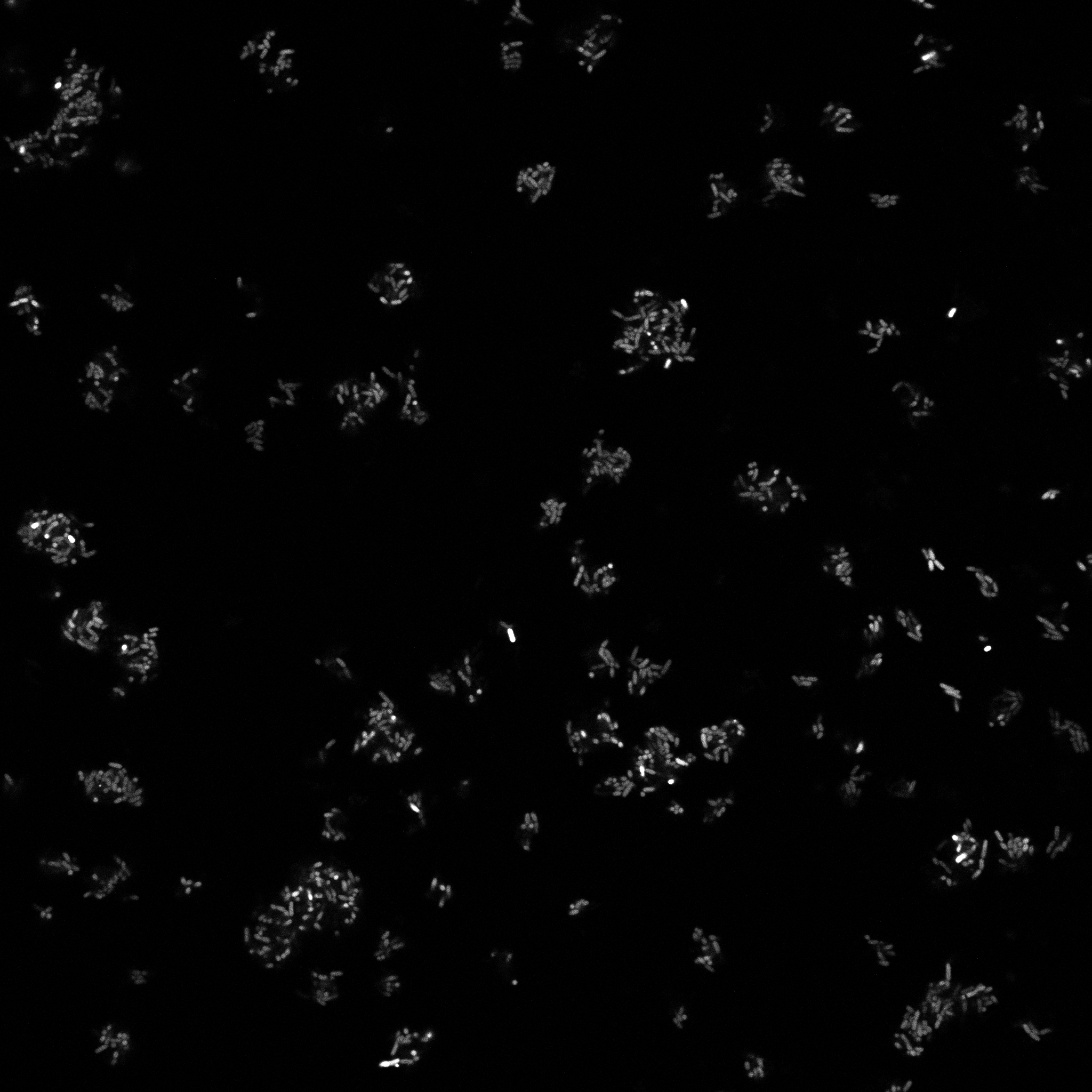

Supplement: Supplementary file 6 — Source data Fig. 5 [file 44320_2026_202_MOESM6_ESM.zip › SD figure 5/5H/t12.tif]

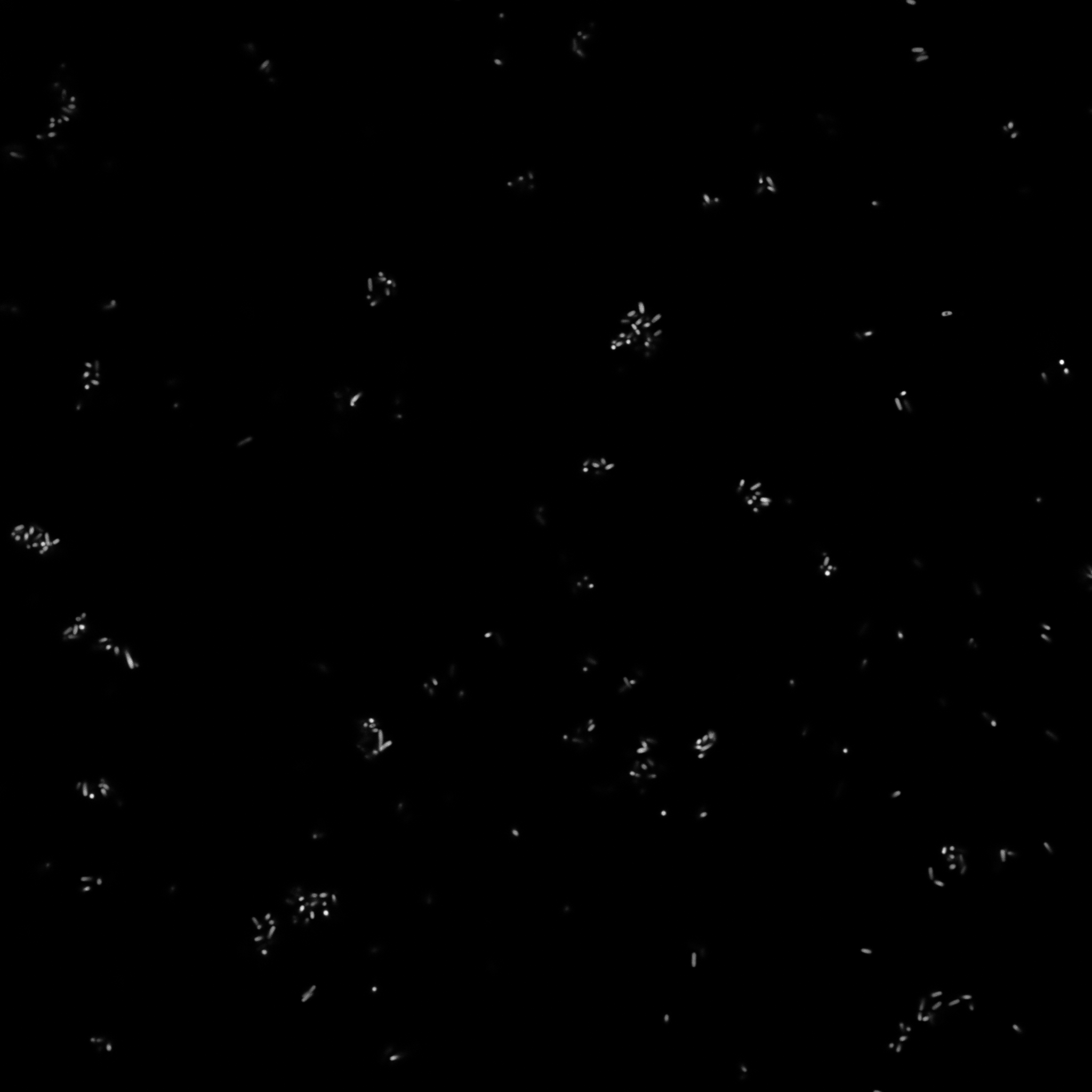

Supplement: Supplementary file 6 — Source data Fig. 5 [file 44320_2026_202_MOESM6_ESM.zip › SD figure 5/5H/t0.tif]

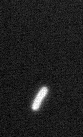

Supplement: Supplementary file 10 — Figure EV2 Source Data [file 44320_2026_202_MOESM10_ESM.zip › SD figure EV2/EV2A/11_09_AverageStack_lin2.tif]
