# Supplementary material for: A universal surface functionalization technique to chemically enhance live microbial cells
Source: Mol Syst Biol. 2026 Mar 16;22(6):962–78. doi: 10.1038/s44320-026-00202-z (PMC13230988; doi:10.1038/s44320-026-00202-z)
Supplement: Supplementary file 11 — Expanded View Figures [file 44320_2026_202_MOESM11_ESM.pdf]

Expanded View Figures

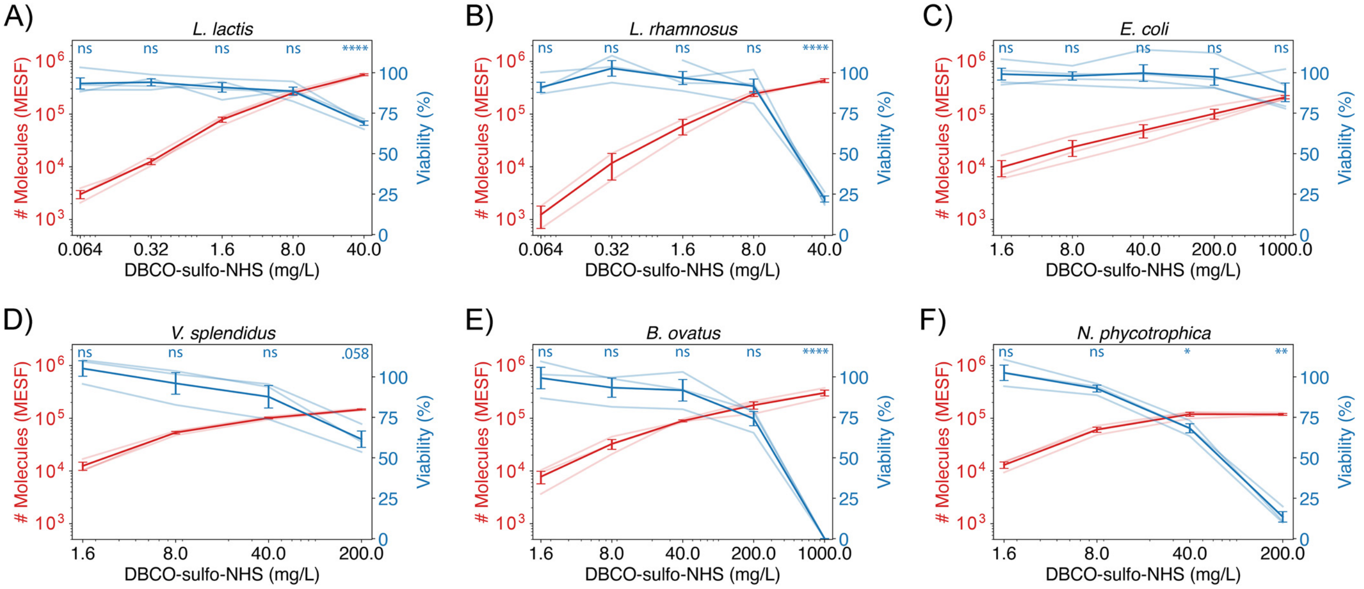

Figure EV1. Quantification of efficiency and viability trade-off for the six strains screened.

(**A-F**) Semi-transparent curves represent different biological replicates. Significance of viability effects are displayed on top of graphs and were calculated with a one-sided, one-sample *t* test with $n = 3$ or 4 depending on the number of biological replicates in the sample. *P* values were corrected using the Benjamini–Hochberg procedure. The meaning of the symbols is $P > 0.1$ (ns), $0.1 > P > 0.05$ (displayed), $0.05 > P > 0.01$ (*), $0.01 > P > 0.001$ (**), $0.001 > P > 0.0001$ (***), and $0.0001 > P$ (****). Source data are available online for this figure.

 © The Author(s)

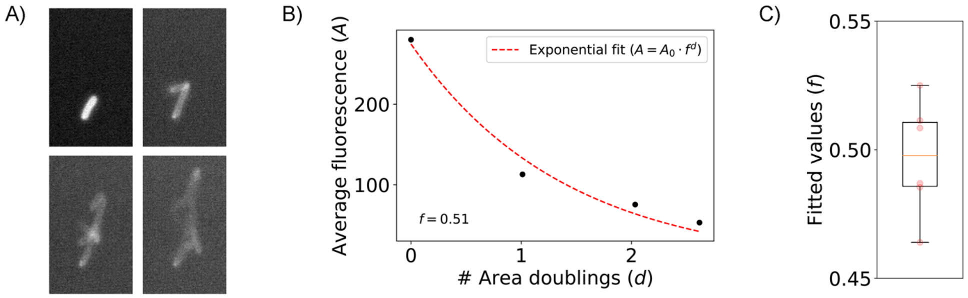

Figure EV2. Quantification of the dilution of surface modification with growth.

(**A**) Microscopy images showing the growth of a lineage of cells functionalized with fluorophores. (**B**) Average cell fluorescence (A) as a function of area doublings ($d = \log_2(\text{area}/\text{initialarea})$). Cells were segmented from images in (**A**) to calculate average fluorescence and area values. An exponential fit was then performed to estimate the fraction of fluorescence retained after each area doubling (f). (**C**) Fraction of fluorescence retained after every doubling (f). A box plot represents the first and third quartiles of six biological replicates with a line at the median and whiskers representing maximum and minimum values. Source data are available online for this figure.

© The Author(s)
